# Exploring Motor Network Connectivity in State-Dependent Transcranial Magnetic Stimulation: A Proof-of-Concept Study

**DOI:** 10.3390/biomedicines12050955

**Published:** 2024-04-25

**Authors:** Laura Marzetti, Alessio Basti, Roberto Guidotti, Antonello Baldassarre, Johanna Metsomaa, Christoph Zrenner, Antea D’Andrea, Saeed Makkinayeri, Giulia Pieramico, Risto J. Ilmoniemi, Ulf Ziemann, Gian Luca Romani, Vittorio Pizzella

**Affiliations:** 1 Department of Neuroscience, Imaging and Clinical Sciences, G. d’Annunzio University of Chieti-Pescara, Via dei Vestini 31, 66100 Chieti, Italy; 2 Institute for Advanced Biomedical Technologies, G. d’Annunzio University of Chieti-Pescara, Via dei Vestini 31, 66100 Chieti, Italy; glromani@itab.unich.it; 3 Hertie Institute for Clinical Brain Research, University of Tübingen, 72076 Tübingen, Germanyulf.ziemann@uni-tuebingen.de (U.Z.); 4 Department of Neuroscience and Biomedical Engineering, Aalto University School of Science, P.O. Box 12200, 00076 Aalto, Finland; 5 Department of Neurology & Stroke, University of Tübingen, 72076 Tübingen, Germany; 6 Department of Psychiatry, Faculty of Medicine, University of Toronto, Toronto, ON M5T 1R8, Canada; 7 Institute for Biomedical Engineering, University of Toronto, Toronto, ON M5S 3G9, Canada; 8 Temerty Centre for Therapeutic Brain Intervention, Centre for Addiction and Mental Health, Toronto, ON M6J 1H1, Canada

**Keywords:** functional connectivity, Motor Network, electroencephalography (EEG), transcranial magnetic stimulation (TMS), motor evoked potential (MEP), corticospinal excitability, brain state

## Abstract

State-dependent non-invasive brain stimulation (NIBS) informed by electroencephalography (EEG) has contributed to the understanding of NIBS inter-subject and inter-session variability. While these approaches focus on local EEG characteristics, it is acknowledged that the brain exhibits an intrinsic long-range dynamic organization in networks. This proof-of-concept study explores whether EEG connectivity of the primary motor cortex (M1) in the pre-stimulation period aligns with the Motor Network (MN) and how the MN state affects responses to the transcranial magnetic stimulation (TMS) of M1. One thousand suprathreshold TMS pulses were delivered to the left M1 in eight subjects at rest, with simultaneous EEG. Motor-evoked potentials (MEPs) were measured from the right hand. The source space functional connectivity of the left M1 to the whole brain was assessed using the imaginary part of the phase locking value at the frequency of the sensorimotor μ-rhythm in a 1 s window before the pulse. Group-level connectivity revealed functional links between the left M1, left supplementary motor area, and right M1. Also, pulses delivered at high MN connectivity states result in a greater MEP amplitude compared to low connectivity states. At the single-subject level, this relation is more highly expressed in subjects that feature an overall high cortico-spinal excitability. In conclusion, this study paves the way for MN connectivity-based NIBS.

## 1. Introduction

For over three decades, non-invasive brain stimulation (NIBS) has been used to modulate brain activity in healthy subjects and patients [1,2,3] for scientific, diagnostic, and therapeutic purposes. However, the effects are highly variable, thus limiting its clinical use [4]. Recently, it has become clear that to reduce the variability of the stimulation effects not only between subjects but also between sessions, the internal state of the brain before NIBS must be taken into account [5,6,7,8]. For this purpose, the integration of NIBS with techniques which is able to non-invasively measure neuronal activity, such as electroencephalography (EEG) [9,10], has offered a window into the state of the brain before the stimulation [11]. This brain state has largely been assessed by looking at the spectral characteristics of the EEG signal at the channels near the stimulation site [12]. In particular, the phase of the sensorimotor 9–13 Hz μ-rhythm has been considered an indicator of cortical excitability that determines the response to transcranial magnetic stimulation (TMS) [11,13,14], although the observed phase effects vary with varying stimulation and analysis parameters [15,16,17].

In parallel to advances in EEG-informed NIBS, neuroscience has seen a paradigmatic shift from a modular view, in which different functional units act as independent processors, to a large-scale network view, in which dynamic interactions between areas of the brain are crucial for cognition and behavior [18]. While functional magnetic resonance studies have been seminal in this regard [19], non-invasive electrophysiology has contributed to this view by the characterization of neuronal networks in terms of their oscillatory fingerprints [20,21,22,23,24,25,26,27], i.e., a view largely supported by the communication through coherence (CTC) hypothesis [28,29,30]. In this framework, a brain state can be described as the evolving dynamics of one or more large-scale networks [31], including the so-called resting-state networks [32], that constrain ongoing activity in the absence of any externally imposed task. Several studies have investigated the effects of invasive and non-invasive stimulation on resting-state networks and, more in general, on remote regions connected to the stimulation site [33,34,35]. However, while the techniques for connectomic neuromodulation studies appear mature [36,37,38,39,40,41], scarce evidence has been provided, so far, for the impact of network dynamics on stimulation effects, explicitly using functional connectivity approaches [42]. In addition, so far, only one study [43] has investigated sensor-level functional connectivity as a feature for brain-state dependent stimulation, although the accuracy in space and time is limited by the sensor-level analysis and by the real-time software implementation.

The aim of this work is to bridge the gap between the study of brain networks with non-invasive electrophysiology and brain state-dependent stimulation, with the long-term goal of systematically using EEG-derived brain networks to drive the stimulation in space and time with millimeter and millisecond resolutions [44,45]. Here, we provide a proof-of-concept study that fast-dynamic brain networks [44] can be derived from combined EEG-TMS data in the pre-stimulation resting period and that the momentary connectivity state of such networks is related to the stimulation endpoint. Specifically, our proof-of-concept study assessed, using data from a previous study [46] and the phase-locking of the oscillatory μ-rhythm, the functional connectivity between the primary motor cortex (M1) signal and the signals at all other brain locations, and its putative relation to the amplitude of motor-evoked potentials (MEPs). The choice of the μ-rhythm frequency for our analysis is driven by the observation that ongoing oscillations in the Motor Network (MN) at rest are expected to synchronize at this frequency [47]. Specifically, we hypothesized that (1) the pattern of long-range functional connectivity of the primary motor cortex (M1) in the pre-stimulation period largely overlaps the spatial topography of the MN; (2) the connectivity state of this network impacts the effect of TMS pulses delivered at M1 on a trial-by-trial basis; and (3) such an impact is augmented if not only connectivity properties but also local properties are considered.

## 2. Material and Methods

### 2.1. Participants and Experiment

Eight right-handed adults (5 females, 3 males; mean ± SD age 23.5 ± 3.3 years) with no history of neurological and/or psychiatric pathologies were enrolled and correctly completed the study. All participants gave written informed consent before participation. The study was approved by the local ethics committee at the University of Tübingen and conducted in accordance with the Declaration of Helsinki. Data were acquired at the University of Tübingen using a concurrent EEG–TMS setup in a single session for each participant (duration about 3 h). EEG and electromyography (EMG) were simultaneously recorded (sampling rate 5 kHz). EEG was recorded using a TMS-compatible 128-channel cap (EasyCap BC-TMS-128, EasyCap, Herrsching, Germany) positioned according to the International 10–5 system. EMG was recorded from the abductor pollicis brevis (APB) and first dorsal interosseous (FDI) muscles of the right hand in a bipolar belly-tendon montage. A TMS stimulator (PowerMAG Research 100, MAG & More, Munich, Germany) was used to deliver biphasic pulses through a figure-of-eight coil (PMD70-pCool, with a 70 mm winding diameter, MAG & More, Munich, Germany). The relative head and coil positions were tracked using optical neuronavigation (Localite GmbH, Bonn, Germany). After the preparation of EEG, EMG, neuronavigation, and pinpointing the EEG electrodes, the hand representation of the left M1 (lM1) was targeted, orienting the coil such that the strongest field was induced in a posterior-lateral to anterior-medial direction. The motor hotspot was defined as the position and orientation of the coil requiring the smallest stimulation intensity to evoke MEPs in either of the two hand muscles. The resting motor threshold (rMT) was defined as the minimum stimulation intensity able to elicit MEPs with peak-to-peak amplitudes > 50 µV in 50% of test pulses [3]. During the experiment, participants were seated comfortably and fixated on a cross located approximately 1 m in front of them. One thousand single TMS biphasic pulses were then applied in a single session with an interstimulus interval of 2 ± 0.25 s at a stimulation intensity of 110% rMT. A different analysis using the same dataset was previously reported [46].

### 2.2. Data Processing

EEG data were down-sampled to 1 kHz and split into windows ranging from −1004 to −4 ms relative to the TMS pulse. A Laplacian-based trend detection was applied to remove slow trends in the data, and noisy or bad channels/trials were identified and removed. A subject-level independent component analysis (ICA) was then performed using the FastICA technique [48] in the subspace generated by the 35 largest principal vectors. Further details are given in [46] and in the preprocessing source code at www.github.com/bnplab/causaldecoding, accessed on 2 October 2021.

For each independent component (IC), the channel-level topography and power spectra were calculated and visually inspected by two experienced researchers; ICs with a clear artifactual hallmark were discarded. The remaining ICs were projected to the source space using the eLORETA spatial filter [49] to identify the corresponding neural generators. ICs whose source space topography showed maxima over the motor cortices were considered for the identification of the individual μ-rhythm frequency, defined as the frequency in the 9–13-Hz range at which a maximum in the IC power spectrum occurs. Among all Ics, only those with a clear peak at the μ frequency and a motor signature in the source space were used to reconstruct channel-level cleaned signals for further analysis. Specifically, Ics used to reconstruct cleaned signals had a ratio of at least 5 between the μ power and beta power in their spectral representation, and when projected to the source space, they were localized in motor areas (e.g., primary motor cortices). This step allowed us to disentangle the contribution of μ-rhythm activity from the original signal.

EMG data were divided into stimulus-locked trials ranging from −500 s to 500 ms relative to the TMS pulse. First, slow drifts were removed by trendline fitting; then, 50 Hz of noise was removed [46]. The visual inspection of EMG data allowed us to remove bad trials, such as those presenting clear EMG activity before the pulse. TMS-related artifacts were removed by relying on an exponential fitting. For each trial and channel, the peak-to-peak MEP amplitude was estimated as the EMG signal range of the manually defined window of data that contain the MEP [46]. Finally, an across-trials principal component analysis was applied to the log-transformed MEP amplitudes estimated from the FDI and APB muscles; the first principal component was used in the subsequent analyses as it explained 99.3 ± 0.5% of the variance (mean and standard deviation across subjects). 

### 2.3. Source Estimation

Source estimation from the cleaned channel-level signals was performed with the FieldTrip toolbox [50] by relying on a source model based on a standard template composed of 15,684 uniformly distributed sources in the Montreal Neurological Institute (MNI) space. A non-linear transformation was applied to realign individual EEG sensor positions to the nearest vertex of the scalp mesh [51]. The geometrical mapping of sources to sensors (namely, the lead field matrix) was derived by solving the electromagnetic forward problem using a 3-shell boundary element model (BEM) between the vertices of the standard template and the realigned electrodes with the conductivity values of the head tissues set to 0.33 S/m for the skin, 0.0041 S/m for the bone, and 0.33 S/m for the brain. The dimensionality of the obtained lead fields was reduced for each voxel by retaining the source orientation, explaining most of the variance. Then, the reduced lead field matrix was used to derive the spatial filter operator by the eLORETA method [49]. Finally, the cleaned EEG signals were projected to the source space with the spatial filter matrix, thus obtaining a time-course for each of the 15,684 sources. 

### 2.4. Connectivity Analysis

A seed-based connectivity analysis was performed based on the reconstructed source’s time-courses. The seed was chosen according to the position of lM1 in the MNI space [−45.9 −9.9 54.6]. The time-courses of the seed and target sources, i.e., all the other 15,683 sources, in the 1 s window prior to the stimulation, were band-pass-filtered around the individual μ-rhythm frequency, with a bandwidth of 2 Hz, using a two-pass fourth-order Butterworth filter. The filtered time-courses were padded at both ends by 64 ms and then transformed into their analytic representations by means of the Hilbert transform. Padding was necessary to reduce the edge effects of the filter and the Hilbert transform. Specifically, padding was performed by applying an autoregressive model (Yule-Walker, order 30) in which coefficients were generated from the filtered time-courses. Given the analytic signals of the seed $\Sigma_{S}\left(f,t\right)$ and of each target $\Sigma_{T}\left(f,t\right)$, we extracted the spectral phases $\phi_{S}\left(f\right)=arg\left\{\Sigma_{S}\left(f\right)\right\}$ and $\phi_{T}\left(f\right)=arg\left\{\Sigma_{T}\left(f\right)\right\}$ where $arg\left\{\cdot \right\}$ represents the argument of a complex-valued number. Finally, the imaginary part of the phase-locking value (iPLV) [52] was estimated as ${iPLV}_{S,T}\left(f\right)=\left|\;\langle I\left\{\;exp\left\{ı\;{\Delta \phi }_{S,T}\left(f\right)\right\}\;\right\}\;\rangle \;\right|$ where $\left|\cdot \right|$ denotes the absolute value, $I\left\{\cdot \right\}$ is the imaginary part of a complex-valued number, $\langle \;\cdot \;\rangle$ indicates expectation value across data epochs and the phase difference ${\Delta \phi }_{S,T}\left(f\right)$ was calculated as ${\Delta \phi }_{S,T}\left(f\right)=\phi_{S}\left(f\right)-\phi_{T}\left(f\right)$. We relied on the iPLV metric because we aimed to characterize connectivity through the phase coupling of neuronal oscillations in line with the CTC hypothesis [29,30] with a robust approach to EEG mixing artifacts [26]. 

The procedure described above led to an individual seed-based functional connectivity map at the μ-rhythm frequency. The group-averaged functional connectivity map was then computed to identify sources functionally connected to the left M1 in the following termed connectivity Regions of Interest (cROIs) that were considered for subsequent analysis.

Of note, we explicitly decided not to a priori select trials with a high signal-to-noise ratio, a procedure employed, e.g., by Zrenner et al. [11], for phase detection to avoid potential biases in connectivity analysis or subsequent analyses.

### 2.5. Relation between Functional Connectivity and Motor-Evoked Potential

To assess if the observed functional connectivity is related to the amplitude of the MEP signal at the individual level, for each of the cROIs, we split the trials into two subsets according to the median of the iPLV values: high-connectivity (HC) and low-connectivity (LC) trials. Then, for each cROI and subject, we calculated the MEP change relative to the mean MEP amplitude for the HC and LC trials separately and determined with a paired sample *t*-test whether a difference in these classes of trials existed.

Additionally, we asked whether a set of trials existed for which, taken together, all cROIs exhibited high or low connectivity to lM1. We termed these subsets as high-connectivity trials for the network (HC_network) and low-connectivity trials for the network (LC_network). The modulation of MEP amplitudes in the HC_network and LC_network trials was assessed, similarly to that between HC and LC trials, by a paired sample *t*-test. 

Control analyses were run for the modulation of MEP in different trial subsets defined at the subject level according to criteria that did not consider functional connectivity as follows: (i) splitting the trials into the first and second part of the recording; (ii) splitting the trials into even and odd. For each of these different trial-splitting approaches, median MEP amplitudes across the first and second subsets were calculated for each subject, and a paired sample *t*-test (two-tails) was run to assess modulations of MEP amplitudes between the two subsets.

### 2.6. Coupling Directionality 

To assess coupling directionality, we relied on the multivariate phase slope index (MPSI) [53], known to be more reliable than the corresponding bivariate approaches [54,55]. The calculation of MPSI is based on the estimation of the cross-spectra among time-courses of the cROIs and time-courses of the voxels surrounding the seed. Specifically, to apply the multivariate directionality metric, we selected all voxels, the distance of which were smaller than 4 mm from the seed (a subset of dimensionality *n*) and from the cROI centroids. Each multivariate time series was then built as a matrix, with the first dimension being *n* and the second being the total data length obtained by concatenating one-second prestimulus data for the HC_network and LC_network trials. These time series were used to calculate MPSI over each pair of frequencies in a range spanning 4 Hz centered at the individual μ frequency and with a 1 Hz frequency resolution. To assess the statistical significance of the observed results, we considered a standardized version of MPSI that allowed us to interpret the ratio between MPSI and its standard deviation across estimation segments (jackknife approach) as a pseudo-Z score. Finally, a group-level Z-score was obtained by averaging the individual pseudo-Z score values multiplied by the square root of the number of subjects to normalize the variance of the averaged pseudo-Z score distribution. Of note, the coupling directionality measured by the MPSI pseudo-Z score could not be directly interpreted as a measure of the coupling strength; rather, it estimates the leader and follower role between a pair of multidimensional signals.

### 2.7. Phase Estimation

The phase of the μ-rhythm signal at stimulation was estimated by following the approach of [11]. Specifically, we extracted the signal 500 ms preceding the TMS stimulation from the lM1 region in the source space, then a forward-backward filter in the individual μ-band (order = 64) was applied and, finally, the filtered signal was trimmed at the beginning and end by 64 ms to remove the edge effects of the filter. An autoregressive model of order 30 was then used to predict the signal from −64 to +64 ms centered around the TMS stimulation. The phase of the signal was then obtained by applying the Hilbert transform to the predicted signal and by extracting the phase at time zero. Of note, the phase values and the connectivity values were estimated for all trials that survived artifact rejection. As mentioned in the previous paragraph, no trials were discarded based on EEG power. 

### 2.8. Linear Regression Analysis 

To investigate the relationship between the long-range Motor Network connectivity, as measured by the phase locking (i.e., phase differences) of μ-rhythm oscillations, and local properties of M1, as measured by the phase of the μ-rhythm oscillation at M1, we tested five different linear models for MEP amplitude prediction at the individual level across all trials. 

First, we tested a model in which connectivity values between lM1 and a single cROI were used as an independent variable $\left[a+b_{1}\;{iPLV}_{lM1,{\;cROI}_{1}}\right]$; then, we added the connectivity between *lM*1 and all other (n−1) cROIs to the first model $\left[a+\sum_{j=1}^{n}b_{j}{iPLV}_{lM1,{\;cROI}_{j}}\right]$. The phase at lM1 was used as the only independent variable in a third model $\left[a+c\cos\varphi +d\sin\varphi \right]$. Then, all the variables were used in a fourth model, including all forms of the connectivity and phase as independent variables $\left[a+\sum_{j=1}^{n}b_{j}{iPLV}_{lM1,{\;cROI}_{j}}+c\cos\phi +d\sin\phi \right]$. In the above equations, ${a,\;b}_{1},\;b_{j},\;c,\;d\;$ are the model parameters, φ is the phase, and ${iPLV}_{lM1,{\;cROI}_{j}}$ is the connectivity between lM1 and all the cROIs. Finally, a constant model was used as the control analysis.

Robust linear regression [56] based on an iteratively reweighted least squares approach implemented in Matlab 2020b (The Matworks Inc., Natick, MA, USA) was used for all models.

The Akaike Information Criterion (AIC) value [57] was calculated to compare these different models. Indeed, the AIC-based model selection weights model performance and complexity in a single metric, and the difference between AICs of different models was an indicator of their relative plausibility [58]. Specifically, we used AIC to answer the question as to whether it is worth adding another variable in the model for the connectivity-based model with one cROI versus the connectivity-based model with all cROIs; the connectivity-based model versus the connectivity and phase-based model; and the phase-based model versus the functional connectivity and phase-based model.

## 3. Results

### 3.1. Functional Connectivity at the μ-Rhythm Frequency Highlights Coupling within the Motor Network 

**Figure 1:**
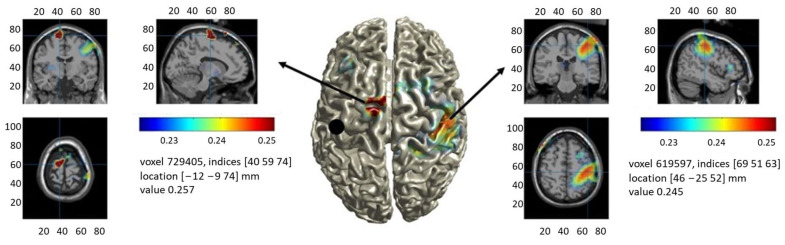
Group-averaged functional connectivity (iPLV) to the left primary motor cortex (lM1, black dot in the left hemisphere of the middle panel). The **middle panel** shows a surface-based projection, and the **left** and **right panels** show two different orthographic projections of the same map. All views highlight that lM1 is functionally connected to the right motor cortex (rM1) and to the left supplementary motor area (lSMA), as defined by the MNI coordinates of their centroids.

EEG preprocessing evidenced that, on average, 23% of the channels and 18% of the trials were contaminated by artifacts and were, therefore, excluded from the following analysis. The average μ-rhythm peak frequency across subjects was 10.5 ± 1.5 Hz. For each subject, a map of iPLV with respect to lM1 was obtained at the individual μ-rhythm peak frequency; the grand average of these individual iPLV maps is shown in Figure 1. The surface-based top view of Figure 1 in the middle panel shows the location of lM1 (black dot), and the regions functionally connected to it are a color-coded representation in which red indicates high connectivity to lM1. The orthographic views of Figure 1 in the left and right panels better show the location for the red spots of Figure 1 in the middle panel and highlight that lM1 is functionally connected to the left supplementary motor area (lSMA, centroid MNI coordinates [−12 −11 74]) and to the right motor cortex (rM1, centroid MNI coordinates [40 −25 52]). While recent EEG reports have detected a correlated pattern of functional connectivity resembling the motor system at broadband [59] and at alpha band [60] frequencies, the current findings indicate the emergence of the Motor Network at the individual μ-rhythm peak frequency in baseline activity preceding TMS. For the following analyses, lSMA and rM1 regions obtained with the described approach were employed as the set of cROIs. 

### 3.2. MEP Amplitude Modulates with Functional Connectivity of the Motor Network 

**Figure 2:**
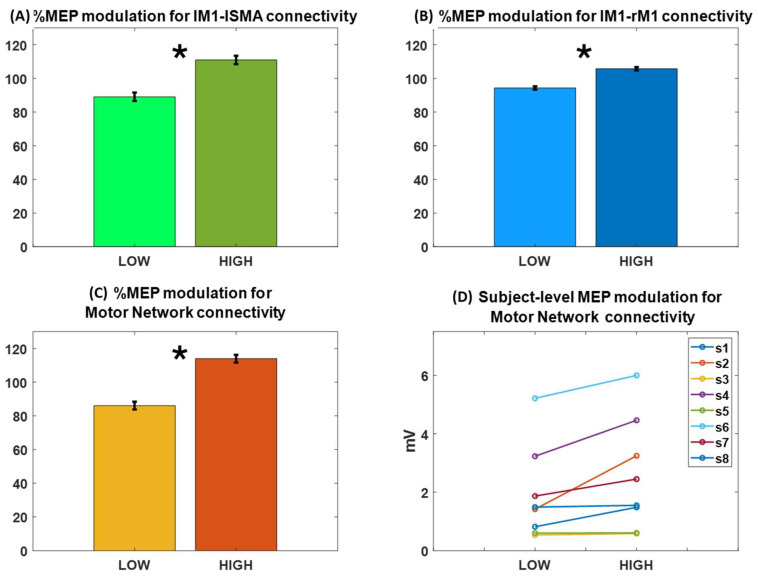
MEP amplitude modulation and percentage values in trials with high (HC) and low (LC) connectivity and median split-based definition. Bars indicate mean MEP amplitude in the corresponding set of trials, which across all subjects and whiskers indicate the standard error of the mean. (**A**) MEP modulation (one-tail paired sample *t*-test, * *p* = 0.03) for connectivity between the left motor cortex (lM1) and the left supplementary motor area (lSMA). (**B**) MEP modulation (one-tail paired sample *t*-test, * *p* = 0.01) for connectivity between lM1 and the right motor cortex (rM1). (**C**) MEP modulation (one-tail paired sample *t*-test, * *p* = 0.01) in the subset of trials in which connectivity between lM1 and lSMA and connectivity between lM1 and rM1 are simultaneously low (LC_network) or high (HC_network). (**D**) A positive modulation of connectivity with MEP amplitude (logarithmic value of MEP amplitudes is shown on the y-axis) was observed in the majority of the subjects.

The modulation of the MEP amplitude in the lM1-lSMA HC trials, defined as the percentage change in the MEP amplitude with respect to the MEP mean value, was calculated for each subject, and the average value and its standard error are shown in Figure 2A together with the modulation in lM1-lSMA LC trials. On average, a difference of 21.8 ± 2.5% (mean ± standard error of the mean) in MEP amplitudes was observed for lM1-lSMA HC trials with respect to lM1-lSMA LC trials (one tail paired-sample *t*-test, *p* = 0.03). This result points towards the spontaneous facilitation effect of SMA on M1 at rest, i.e., a high lM1-lSMA connectivity enhances MEP amplitude in line with the facilitation obtained by conditioning M1 by stimulating SMA with a cortico-cortical-paired associative stimulation protocol [61].

Similarly, Figure 2B shows that modulation of 11.4 ± 1.0% is featured by an MEP amplitude in the lM1-rM1 HC trials with respect to lM1-rM1 LC trials (one tail paired-sample *t*-test, *p* = 0.01). It should be noted that, in general, lM1-lSMA HC trials are a different subset than lM1-rM1 HC trials similar to LC trials and that the facilitatory effect of lM1-rM1 connectivity found here is smaller in extent with respect to the lM1-lSMA effect, which is in line with our observation that the overall functional connectivity between lM1 and rM1 is weaker than that of lM1 with lSMA.

Figure 2C shows the MEP modulation in the subset of trials in which lM1-lSMA and lM1-rM1 functional connectivity trials are both above or below their corresponding median level. The overlap between HC trials for lM1-lSMA and that for lM1-rM1, across all subjects, was 57% (median value) with an interquartile range of 7%, meaning that the number of trials in which the consistent high or low coupling of the whole network (HC_network or LC_network trials) is observed is above the chance level. A significant increase of about 27.9 ± 2.3% for MEP in the HC_network trials was observed with respect to LC_network trials (one-tail paired-sample *t*-test, *p* = 0.01). The network-level MEP modulation was, thus, increased by about 28% with respect to the largest modulation observed for single node pairs, i.e., lM1-lSMA connectivity.

Finally, Figure 2D shows the modulation of MEP in the HC_network and LC_network at the individual level for the eight subjects. While for some of the subjects, the positive relation was more evident, others did not show a clear effect. Interestingly, the subjects that show weak or no effect are those that feature low cortico-spinal excitability, as indicated by their low MEP amplitude values (subj3, subj5, subj8).

Notably, no significant difference was observed between MEP amplitudes in the first half versus the second half of the trials in the recording (paired-sample *t*-test, *p* = 0.20). This analysis allowed us to rule out possible habituation or potentiation effects induced by the high number of stimuli delivered in each experimental session. Similarly, MEP amplitudes in even versus odd trials were not significantly different (paired-sample *t*-test, *p* = 0.88), thus allowing to exclude chance effects induced by the trial’s splitting procedure. Finally, we calculated functional connectivity between lM1 and lSMA and between lM1 and rM1 and their relation to MEP amplitudes for the theta (average value across subjects: 5.0 ± 0.7 Hz) and beta (average value across subjects: 21 ± 3 Hz) frequencies. These analyses revealed no significant effect of functional connectivity between lM1 and lSMA or between lM1 and rM1 on MEP amplitude modulation for theta (lM1-lSMA: one tail paired-sample *t*-test, *p* = 0.10; lM1-rM1: one tail paired-sample *t*-test, *p* = 0.23) and beta (lM1-lSMA: one tail paired-sample *t*-test, *p* = 0.47; lM1-rM1: one tail paired-sample *t*-test, *p* = 0.09) frequencies.

### 3.3. Coupling Directionality Reveals the Top-Down Control of SMA on Bilateral M1

The MPSI analysis revealed that in the high functional connectivity trials, coupling directionality, averaged across subjects, indicates connectivity from lSMA to lM1 (pseudo-Z = −3.61, *p* = 3 × 10^−4^) and to rM1 (pseudo-Z = −2.53, *p* = 0.01). Conversely, no significant directionality was assessed for the connectivity between lM1 and rM1 (pseudo-Z = −1.88, *p* = 0.06). Similar results were obtained for low connectivity trials (lSMA-lM1 pseudo-Z = −4.78, *p* = 2 × 10^−6^; lSMA-rM1 pseudo-Z = −2.48, *p* = 0.01; lM1-rM1 pseudo-Z = −1.21, *p* = 0.23). 

### 3.4. A Linear Regression Model That Relies on Network Connectivity and the lM1 Phase Best Predicts MEP

The results for the comparison between a linear regression model at the single subject level, in which the MEP amplitude is predicted only by the functional connectivity between lM1 and lSMA, and a model in which the functional connectivity of lM1-rM1 is added as a second independent variable (i.e., Motor Network model), are reported in the Supplementary Material Tables S1 and S2. These data indicate that the Motor Network model performs overall better than the lM1-lSMA model.

In the following, we further compare, in terms of their respective AIC values, the Motor Network model with different linear regression models in which the MEP amplitude is predicted only by the phase at lM1 (Table 1, column 3), as a model in which MEP is predicted by network-level functional connectivity (Table 1, column 4), and a model in which both are used as independent variables to predict MEP (Table 1, column 5). Additionally, the AIC of MEP prediction using a constant model is reported (Table 1, column 2). Column 6 of Table 1 indicates the preferred model according to the criteria defined in (Burnham and Anderson, 2004 [58]).

Overall, the model with the Motor Network and lM1 phase as independent variables was preferred in 4 out of 8 subjects, while a model with the Motor Network only as an independent variable was preferred in 2 out of 8 subjects. In the remaining 2 subjects, either the phase-only model or the constant model are preferred. 

Table 2 shows the plausibility of all the tested models.

Overall, Table 1 and Table 2 indicate that including local (lM1 phase) and long-range (Motor Network) characteristics of the source space EEG signal results in the best or a plausible predictive model for single-trial MEP amplitudes. 

## 4. Discussion

In the present proof-of-concept study, we show that the EEG-derived long-range connectivity of the primary motor cortex in the pre-stimulation period at an individual μ-rhythm peak frequency is largely congruent with the Motor Network and that the connectivity state of this network modulates the motor responses evoked by the transcranial magnetic stimulation of the primary motor cortex. Specifically, the stronger coupling of left M1 with the left supplementary motor area and right M1, measured by the phase locking of μ-rhythm oscillations, was related to larger motor-evoked potential amplitudes and vice versa. These findings indicate that corticospinal excitability is associated with the coordinated interaction among key areas of the Motor Network rather than only with the local activity of M1. Importantly, the observed positive relation between Motor Network connectivity and MEP amplitudes holds at the individual level, even if not for all subjects. Subjects that do not show the effect feature an overall low cortico-spinal excitability, as indexed by low MEP values, across the whole recording. Thus, we speculate that this lack of modulation is due to a generally low responsiveness and not to the relation to Motor Network connectivity. 

Previous work investigating an association between the MEP amplitude and pre-stimulus EEG phase-locking, as measured by the coherence magnitude, observed a coupling between the stimulated primary motor cortex and a large swath of the centro-parietal cortex in the delta band and the frontal cortex in the high beta band [42]. Yet, the present findings indicate that brain connectivity states affect corticospinal excitability in a topographically selective (i.e., Motor Network) fashion. Our result was likely obtained using a source-level functional connectivity approach based on a metric robust-to-field spread and volume conduction effects [26] compared to the approach used by [42]. The more recent study from Vetter et al. [43] investigated, in a real-time EEG-TMS experiment, the association between the MEP amplitude and pre-stimulus EEG phase-locking between two specific EEG channels (after the application of a Hjorth montage) located approximately above the motor cortices. Overall, this study concludes that functional connectivity was predictive of cortico-spinal excitability together with power and phase. Nevertheless, this study employs seed-based sensor-level connectivity analysis, which makes it impossible to assess whether the considered signals actually come from motor areas. Similarly, the real-time setting available for the experiment did not allow us to consider more than two channels and, thus, to investigate the potential augmentation of the observed effect when more than just two regions in a network are connected.

The positive relationship between MEP amplitude and pre-stimulus Motor Network connectivity is consistent with the neurophysiological and neuroimaging lines of evidence from functional Magnetic Resonance Imaging, indicating that such functional interactions are relevant for MEP [62] and for hand function in healthy individuals [63]. Clinically, it has been observed that intra-hemispheric M1-SMA [64,65] and inter-hemispheric M1-M1 [64,66] functional connections are behaviorally relevant for recovery after motor stroke as well as predictive of the functional improvement induced by a repetitive TMS stimulation [67,68]. Moreover, our results for coupling directionality within the Motor Network indicate an overall stable intrinsic coupling directionality from lSMA to lM1 and rM1, which is in line with previous evidence of SMA’s conditioning effect on M1 excitability [61] as well as with dynamic causal modeling-based or Granger-causality SMA control over primary motor cortices [63,69].

In addition, the lack of dominant directionality between the two motor cortices in the resting brain is in line with Grefkes et al. [70].

Furthermore, our individual analysis results show that, for the majority of the subjects, a model including both long-range phase-locking within the Motor Network and the phase of M1 oscillation results in a better prediction of MEP amplitudes than either one of these factors alone. A model based on Motor Network connectivity alone is, in general, more plausible than a model based on the M1 phase alone. Of note, in contrast to previous works investigating the role of the phase in predicting MEP amplitudes, e.g., [11], we did not select trials on a power-based criterion; this might justify the difference between our results and previous ones for phase-based prediction.

Finally, our findings pair to the work by Stefanou et al. [71] in supporting the idea that functional connectivity can be directly exploited to design-paired with multi-coil stimulation protocols in which the stimulation is delivered when nodes in the network are phase-coupled. Indeed, the functional connectivity approach used in our paper can be extended to real-time estimation [72,73,74] and, thus, translated into protocols for state-dependent connectivity-based stimulation with the ability to influence post-stimulation-evoked responses [75].

It should be noted that functional connectivity before the stimulation might be influenced by the previous pulse (or pulses) in a way that is still debated in the literature. Post-stimulation increases have been observed in Pieramico et al. [35], while a reduction in global EEG connectivity was observed in the healthy subject’s cohort of Vlachos et al. [76]. Nevertheless, even if the observed Motor Network connectivity pattern was influenced by the previous stimulation, this still represents the actual connectivity state of the network, which we found to be related to the MEP amplitude.

Although we acknowledge that a limitation of this study is the limited size of our cohort, it must be noted that our data rely on a high number of trials for each subject (with the overall number of trials at around 8000). The final aim of our study was to provide a proof-of-concept of the possibility of extracting an EEG-based Motor Network from EEG-TMS data, as well as to assess the relationship between Motor Network connectivity and cortico-spinal excitability at a single subject level and to possibly take advantage of our results for individualized connectivity targeted stimulation. For this reason, relying on many trials per subject allowed us to perform such an investigation in a robust manner in this study in which a subset of trials with high or low functional connectivity had to be considered for these purposes. A further limitation of our study is that we only checked for a linear relationship between brain state features and MEP amplitude. Although we acknowledge that non-linear effects might provide additional information to derive innovative brain-state-dependent stimulation protocols, their study is beyond the scope of this work.

## 5. Conclusions

To the best of our knowledge, this is the first study that investigates to what extent the connectivity state of a brain network in source space prior to transcranial magnetic stimulation influences its outcome. Here, we specifically addressed this question for the network linked to the left primary motor cortex at the individual peak frequency of the sensorimotor μ-rhythm. We demonstrate that a high-connectivity state within this network, which largely overlaps with the Motor Network topography, features a facilitation effect on the amplitude of the motor-evoked potential induced by left primary motor cortex stimulation. Notably, the increase in MEP amplitude with enhanced Motor Network connectivity supports the idea that connectivity-informed real-time state-dependent stimulations may have a high potential, including therapeutic efficacy.

## Figures and Tables

**Table 1:** Akaike Information Criterion (AIC) for the tested models.

Subject #	AIC Constant Model	AIC lM1 Phase	AIC Motor Network	AIC Motor Network and lM1 Phase	Preferred Model
**1**	2106, 5	2004, 8	2071, 6	1971, 0	Motor Network and lM1 phase
**2**	1416, 8	1414, 6	1299, 5	1298, 6	Motor Network and lM1 phase
**3**	1724, 4	1716, 9	1726, 9	1719, 1	lM1 Phase
**4**	1867, 0	1863, 6	1858, 4	1855, 5	Motor Network and lM1 phase
**5**	1931, 0	1933, 8	1932, 9	1935, 7	Constant
**6**	1823, 4	1830, 1	1822, 4	1824, 8	Motor Network
**7**	2178, 2	2180, 3	2173, 6	2176, 2	Motor Network
**8**	1626, 0	1622, 9	1602, 1	1597, 7	Motor Network and lM1 phase

**Table 2:** Plausibility of the tested models.

Subject #	Constant Model	lM1 Phase	Motor Network	Motor Network and lM1 Phase
**1**	Not plausible	Not plausible	Not plausible	Preferred
**2**	Not Plausible	Not Plausible	Plausible	Preferred
**3**	Not plausible	(Mildly) Preferred	Not plausible	Plausible
**4**	Not Plausible	Not Plausible	Plausible	Preferred
**5**	Preferred	Plausible	Plausible	Plausible
**6**	Plausible	Not Plausible	Preferred	Plausible
**7**	Not Plausible	Not Plausible	Preferred	Plausible
**8**	Not plausible	Not plausible	Not plausible	Preferred

## Data Availability

Data are available upon request. There is no consent from the study participants to make the data publicly available.

## Supplementary Materials

The following supporting information can be downloaded at: https://www.mdpi.com/article/10.3390/biomedicines12050955/s1, Table S1: Model comparison; Table S2: Model plausibility.
